# Selenium and Redox Enzyme Activity in Pregnant Women Exposed to Methylmercury

**DOI:** 10.3390/antiox11112291

**Published:** 2022-11-19

**Authors:** Vasco Branco, Luís Carvalho, Cássia Barboza, Eduarda Mendes, Afonso Cavaco, Cristina Carvalho

**Affiliations:** 1 Research Institute for Medicines (iMed.ULisboa), Faculty of Pharmacy, Universidade de Lisboa, Av. Professor Gama Pinto, 1649-003 Lisbon, Portugal; 2 Centro de Investigação Interdisciplinar Egas Moniz (CiiEM), Instituto Universitário Egas Moniz (IUEM), Quinta da Granja, 2829-511 Monte de Caparica, Portugal

**Keywords:** selenium, selenoproteins, pregnant women, thioredoxin reductase, glutathione peroxidase, thioredoxin, mercury, redox systems

## Abstract

Selenium (Se) is a micronutrient with essential physiological functions achieved through the production of selenoproteins. Adequate Se intake has health benefits and reduces mercury (Hg) toxicity, which is important due to its neurotoxicity. This study determined the Se status and redox enzyme, including selenoproteins’, activity in pregnant women highly exposed to Hg (between 1 to 54 µg Hg/L blood) via fish consumption. A cross-sectional study enrolling 513 women between the first and third trimester of pregnancy from Madeira, Portugal was conducted, encompassing collection of blood and plasma samples. Samples were analyzed for total Se and Hg levels in whole blood and plasma, and plasma activity of redox-active proteins, such as glutathione peroxidase (GPx), thioredoxin reductase (TrxR) and thioredoxin (Trx). Enzyme activities were related to Se and Hg levels in blood. Se levels in whole blood (65.0 ± 13.1 µg/L) indicated this population had a sub-optimal Se status, which translated to low plasma GPx activity (69.7 ± 28.4 U/L). The activity of TrxR (12.3 ± 5.60 ng/mL) was not affected by the low Se levels. On the other hand, the decrease in Trx activity with an increase in Hg might be a good indicator to prevent fetal susceptibility.

## 1. Introduction

Selenium (Se) is an essential nutrient in human biology [1], with recommended intake (see [2] for review) set between 60–75 µg/day for men and 60–70 µg/day for women [3,4,5,6]. Pregnant women are advised to increase Se intake so as to reach a daily surplus of 2–4 µg of Se per day [7].

Selenium intake is very variable worldwide, with a mean value of 40 µg/day in Europe and 93 µg/day in the USA. Populations in seleniferous areas in China have intake values over 1000 µg/day [7], reaching values of 3200 µg/L in blood [8] which can lead to selenosis [6]. Curiously, the lowest Se intake values (3.0 µg/day) have also been reported in China in areas where Keshan disease is endemic [7,9,10].

An adequate Se intake has been associated with several health benefits [11,12,13,14,15,16,17] including a rather controversial protective effect against cancer (see [18] for a review on this topic). Benefits on the cardiovascular system have been reported, albeit epidemiological studies have shown contradictory results [19,20]. On the other hand, supplementation with selenomethionine has been associated with a small increase in the incidence of Type 2 diabetes as seen in men enrolled in the SELECT trial [21]. The toxicity associated with supranutritional Se intake results from its ability to redox cycle with O_2_, generating high levels of reactive oxygen species [22].

**Figure 1:**
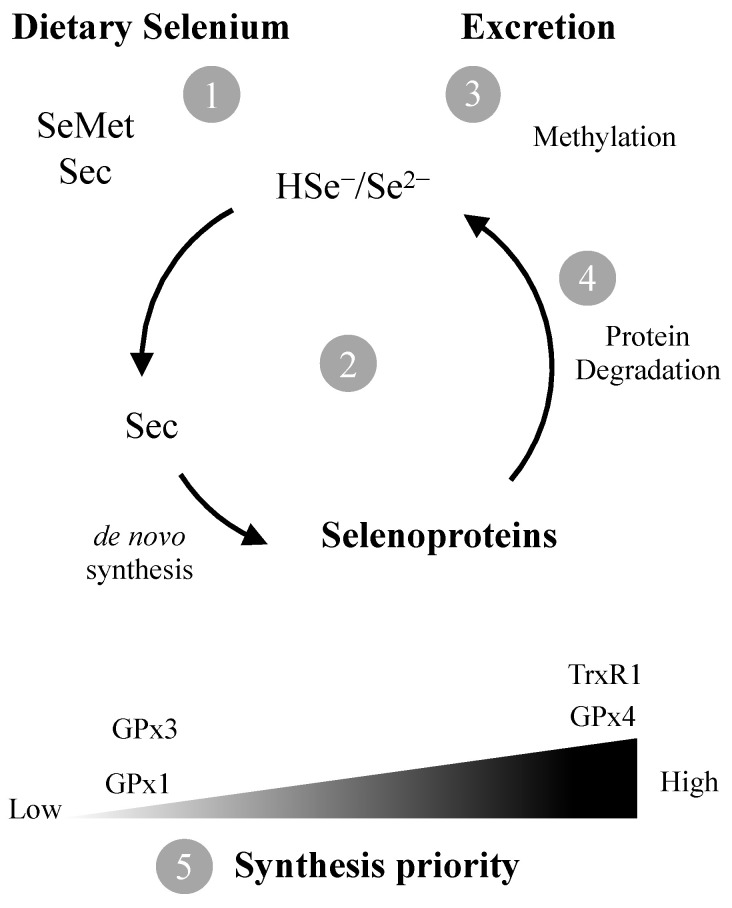
The amino acids selenocysteine (Sec) and selenomethionine (SeMet) are the predominant forms of Se in foods. Following ingestion, they need to be converted (1) to selenide (Se^2–^) which is channeled to *de novo* synthesis of Sec and selenoproteins (2) or methylated for excretion (3). Degradation of selenoproteins results in additional Se^2–^ availability (4). If Se levels are sub-optimal, synthesis of housekeeping selenoproteins such as TrxR1 and GPx4 is prioritized in detriment of ROS scavenging GPx1/3 (5).

Gastrointestinal absorption of Se from food is rapid, but Se compounds must be converted to inorganic selenide (Se^2–^/HSe^–^) before being used in selenoprotein synthesis [22,23] (Figure 1). Selenoproteins are responsible for the main physiological functions of Se and contain at least one selenocysteine (Sec) residue [24]. The low pKa of the selenol group (SeH) confers a high reactivity to Sec and thus, several selenoproteins are enzymes involved in redox reactions [25] with an essential role in cellular and tissue homeostasis [26,27,28].

The selenoenzyme thioredoxin reductase (TrxR) is part of the thioredoxin system, which also comprises thioredoxin (Trx) and NADPH. TrxR is responsible for catalyzing redox reactions in cell growth and antioxidant defense. Trx has a dithiol active site responsible for the reduction of -SH groups of several proteins. Its oxidized form is reduced by TrxR, with NADPH working as an electron donor. This cycle is the basis for the thioredoxin system function [25,29,30]. 

Glutathione peroxidase (GPx) is ubiquitously distributed in all tissues and catalyzes the glutathione-dependent reduction of peroxides, serving an important antioxidant function. Several isoforms exist (see [31] for review) with GPx3 being the one present in plasma. According to WHO recommendations [7], a plasma Se level of 63.2 µg /L is sufficient to optimize plasma GPx activity, while others situated this requirement at 90–100 µg/L [32,33]. 

The relation between selenoproteins’ activity and plasma Se may vary due to several factors, such as the existence of polymorphisms in selenoproteins [27] and exposure to metals interfering with Se bioavailability and selenoprotein activity, such as mercury (Hg) [28,34]. Indeed, Se is known to reduce Hg toxicity by forming insoluble HgSe complexes [28] but, on the other hand, mercury reduces Se bioavailability by interacting with selenoproteins such as Selenoprotein P and selenoproteins with critical function [35,36].

In fact,, redox-active selenoproteins such as TrxR are primary targets for Hg toxicity as shown in several in vitro and in vivo studies [37,38,39,40,41,42], albeit data in human populations are lacking. 

Additionally, very few studies report the relation between Se levels and TrxR in humans, despite many studies in animal models (e.g., [38,43,44]). Moreover, the majority of studies in humans concern groups of patients with very specific pathologies (e.g., [45,46,47]).

Therefore, the aim of this cross-sectional study was to evaluate Se, selenoproteins (TrxR and GPx) and Trx levels in blood and plasma samples from a population of pregnant women living on the Island of Madeira, Portugal, which is traditionally highly exposed to MeHg via fish consumption. The results were subject to statistical analysis and have shown different activity patterns for the redox proteins evaluated.

## 2. Materials and Methods

### 2.1. Study Population Recruitment and Sample Collection

Between 2012 and 2014, a cross-sectional study was conducted encompassing collection of whole blood samples (5 mL) from 513 pregnant women between the 1st and 3rd pregnancy trimester in public healthcare services from Madeira archipelago or before delivery at Hospital Nélio Mendonça. The healthcare units are integrated under the Serviço de Saúde da Região Autónoma da Madeira, E.P.E, SESARAM, hospital centre. Population characteristics are summarized in (Table 1).

Following collection, samples were stored in heparinized tubes. A fraction (2 mL) of some samples (*n* = 167) was centrifuged at 2000 g for 10 min, after which the plasma and leucocyte fractions were collected and mixed. Following collection and processing, blood and plasma samples were frozen at −20 °C until analysis. Hospital Dr. Nélio Mendonça, in Funchal, centralized all samples arriving from other public healthcare units in the archipelago before shipment for analysis at the Faculty of Pharmacy, University of Lisbon. All participants in the study were volunteers and were duly informed about the study protocol and requirements and provided written informed consent before testing. The study protocol was approved by the Ethics Commission for Clinical Research of SESARAM; EPE. (PARECER Nº27/2012 for the project “Avaliação da exposição ao metilmercúrio por consumo de peixe em Portugal: Relação benefício-risco para a saúde infantil” (In Portuguese)). 

### 2.2. Reagents

Sodium selenite; Hydrochloric acid 37% (AppliChem Panreac) for analysis, ACS, ISO; Sodium borohydride (Fluka); Sodium hydroxide pellets (Merck); Hydrogen peroxide solution (Fluka) Analytical; Nitric acid 65% (AppliChem Panreac).

### 2.3. Sample Pre-Treatment for Total Se Analysis

Prior to treatment, all samples were defrosted in an Atom 80 agitator and stirred in a Maxi Mix II Thermolyne vortex mixer. Subsequently, aliquots (250 μL) of whole blood and plasma were transferred to 20 mL glass tubes, followed by addition of 2.5 mL of nitric acid and 750 μL of hydrogen peroxide. The mixtures were stirred and heated in a Dri-Block DB-3D Techne heater at 50 °C for 2 h for digestion. Afterwards, in order to reduce all selenium to Se (IV), 5 mL of hydrochloric acid was added to each tube and the digested samples were heated at 90–95 °C for 40 min and left to cool at room temperature. Volumes were adjusted to 10 mL using volumetric flasks and samples were stored in plastic tubes until analysis. All glass material was submerged in nitric acid 15% for at least 12 h and rinsed thoroughly with Milli-Q Water (18.2 MΩ.cm) prior to use.

### 2.4. Selenium Quantification

Total Se analysis was performed in at least two replicates of each of the whole blood (*n* = 513) and plasma (*n* = 167) samples in a PerkinElmer Instruments AAnalyst 700 atomic absorption spectrometer coupled with a PerkinElmer FIAS 100 flow injection system hydride generator and a PerkinElmer AS 90 autosampler. A freshly prepared solution of 0.2% (*w*/*v*) sodium borohydride dissolved in 0.05% (*w*/*v*) sodium hydroxide, was used for hydride generation [48].

**Figure A1:**
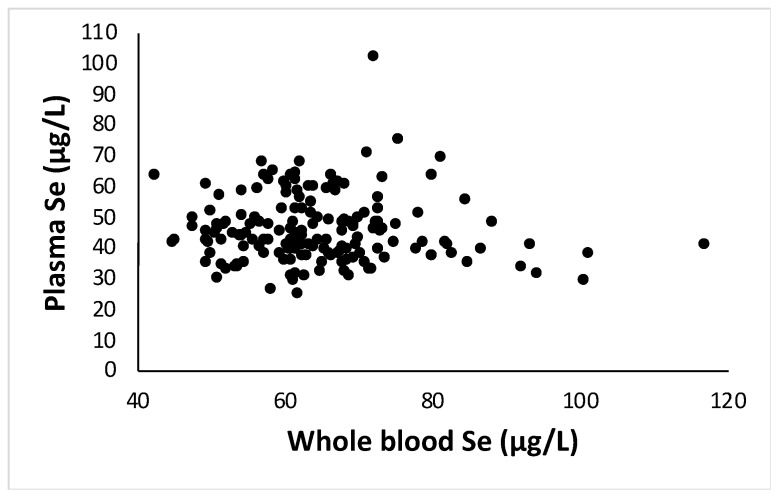
Selenium levels (µg/L) in whole blood versus Se levels in plasma as measured by HG-AAS in samples of 167 women enrolled in the study. The ratio between Se in plasma and whole blood was 73.3 ± 21 per cent.

Since the amount of plasma samples was limited, it was mostly used for analysis of enzymatic activities. Therefore, Se levels were only analyzed in a subset of plasma samples to confirm if the ratio between plasma and whole blood Se was as reported in the literature (A, Figure A1). The ratio between Se in plasma and Se WB averaged 73.3 ± 21 per cent.

### 2.5. Quality Control

The sensitivity of the method was assessed by comparing the signal intensity of a 10 µg/L Se solution, prepared from a reference stock solution (1000 ppm), with the signal intensity recommended by Perkin Elmer (A = 0.200) [48]. The analysis method proved linear in the range 1 to 7.5 ppb. 

Evaluation of precision considered two parameters, repeatability and intermediate precision. To evaluate the repeatability of the method, a standard solution was prepared and analyzed six times. The results showed a relative standard deviation of less than 2%. To evaluate the intermediate precision of the method, a blood sample was analyzed in triplicate on three separate days. The samples were subjected to the same digestion process as the biological samples in the study. The results showed a relative standard deviation below 10%.

The accuracy of the method was tested by analyzing a solution prepared from a reference standard solution. The measurement performed in this solution was within the accepted interval for the reference solution in use.

### 2.6. Thioredoxin Reductase and Thioredoxin Activity in Plasma

TrxR and Trx activities in plasma (*n* = 471) were determined according to the insulin end-point assay described by Árner and Holmgren [49] for complex biological samples. For TrxR activity determination, samples (15 µL) were incubated for 20 min at 37 °C in 96 well plates. Each well contained 0.3 mM of insulin, 660 µM of NADPH, 3 mM of EDTA and 3 µM of Trx (IMCO Corp., Sweden)—previously reduced with DTT at 37 °C and desalted in a NAP-5 column in 85 mM HEPES buffer (pH 7.6). Control wells containing the same reagents excluding Trx addition, were prepared in parallel. After the incubation period, 250 µL of a 1 mM DTNB solution in 6 M guanidine hydrochloride (prepared Tris 200 mM, pH 8.0) were added to each well and absorbance was measured in a microplate reader (Zenyth 3100, Anthos Labtec Instruments) at 412 nm. TrxR activity was quantified as the difference in absorbance between the Trx containing well and the control well. The determinations of Trx activity followed the same procedure used for TrxR, with samples incubated with 100 nM of rat recombinant TrxR. Trx and TrxR were both expressed as ng enzyme/mL plasma. The specific activities of Trx and TrxR in controls were determined from a calibration curve performed using purified Trx (12 kDa) and TrxR (112 kDa; 5 U/mL), respectively. 

### 2.7. Glutathione Peroxidase Activity in Plasma

The activity of cytosolic seleno-dependent GPx was measured in 211 plasma samples according to the method (basic protocol) described by Esworthy and co-workers [50]. Samples (10 µL) were mixed in 96-well plates with sodium phosphate buffer (50 mM, pH 7.0), GSH (10 mM), NADPH (2 mM), sodium azide (1.125 mM) and GR (100 U/mL). A solution of 5 mM H_2_O_2_ was used as the substrate. The decay in absorbance at 340 nm resulting from NADPH consumption was followed for 5 min to calculate enzyme activity. Plasma GPx activity was expressed as units of GPx per liter (U/L), with 1 U equivalent to 1 µmol of NADPH oxidized per minute. 

### 2.8. Analysis of Total Hg in Blood Samples

**Figure 2:**
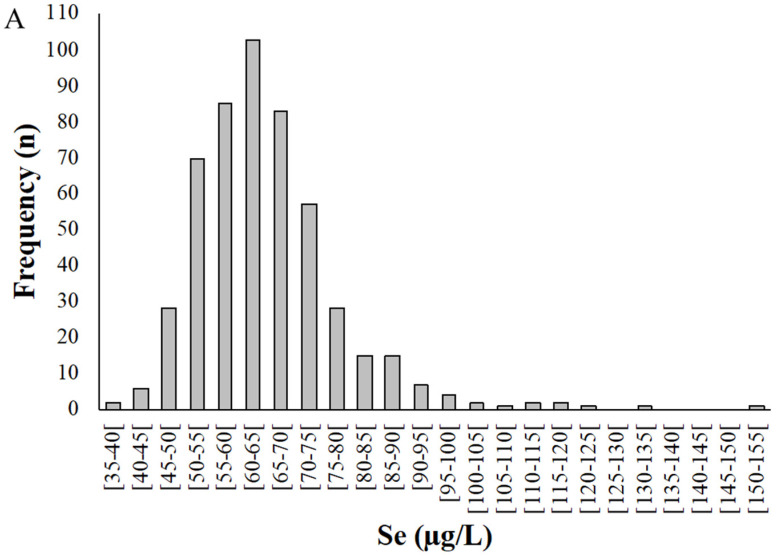

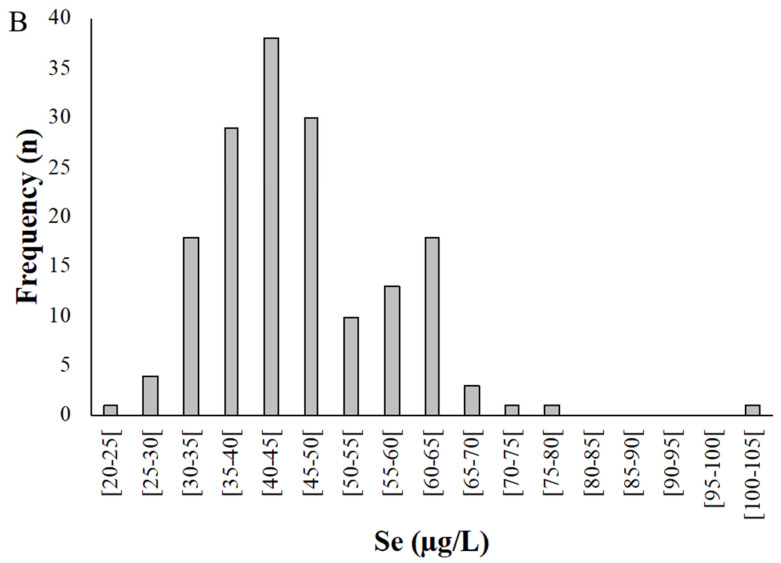
Distribution of selenium levels in whole blood (**A**) and plasma (**B**) samples donated by pregnant women from the Madeira Archipelago. Analyses were carried out by HG-AAS as described in materials and methods.

Results for Total Hg in the same blood samples from the same women enrolled in this study were previously reported and discussed by our group (see [51] for details on results and Hg analysis). For the purpose of the present manuscript and analysis of relations with enzymatic activities, we have grouped women according to their blood Hg level and risk index: <2 µg Hg/L corresponding to women with little or no fish consumption; 2–5 µg Hg/L corresponding to women with moderate fish intake; 5–10 µg Hg/L indicating women with a frequent consumption of fish but with a risk index (RI) below 1; 10–20 µg Hg/L corresponding to women with a high risk of exposure (1 < RI < 2); and 20–50 µg Hg/L for women with an RI > 2. Since a very significant positive correlation (Spearman Correlation rank: 0.90; *p* < 0.001;(A, Figure 2A) was observed between Hg in blood and plasma over a broad range of Hg levels, Hg in whole blood was used to evaluate relationships with enzymatic activities since the number of plasma samples available for Hg analysis was limited.

### 2.9. Statistical Analysis

Concentration and activity values were registered in a statistical IBM SPSS v25 database for all samples. Descriptive tests included distribution frequencies and boxplot graphs cross tabulating 2 variables. Non-parametric correlations (Spearman correlation coefficient—rho) and simple linear regression models were estimated for pairs of variables with plausible biological relation. All calculations used a significant level of *p* < 0.05.

## 3. Results

### 3.1. Selenium Concentration in Whole Blood and Plasma

Selenium levels in whole blood samples (Figure 2) ranged from 38.8 to 152 µg/L, with a mean of 65.0 ± 13.1 µg/L (Figure 2A). Among the pregnant women studied, 95% presented a Se concentration in whole blood between 40 and 90 µg/L.

Selenium concentration in plasma (*n* = 167) varied between 24.9 and 102 µg/L, with a mean of 46.1 ± 11.1 µg/L (Figure 2B). On average, Se levels in plasma were 73.3% of the value for whole blood.

### 3.2. Redox Proteins Activity

**Figure 3:**
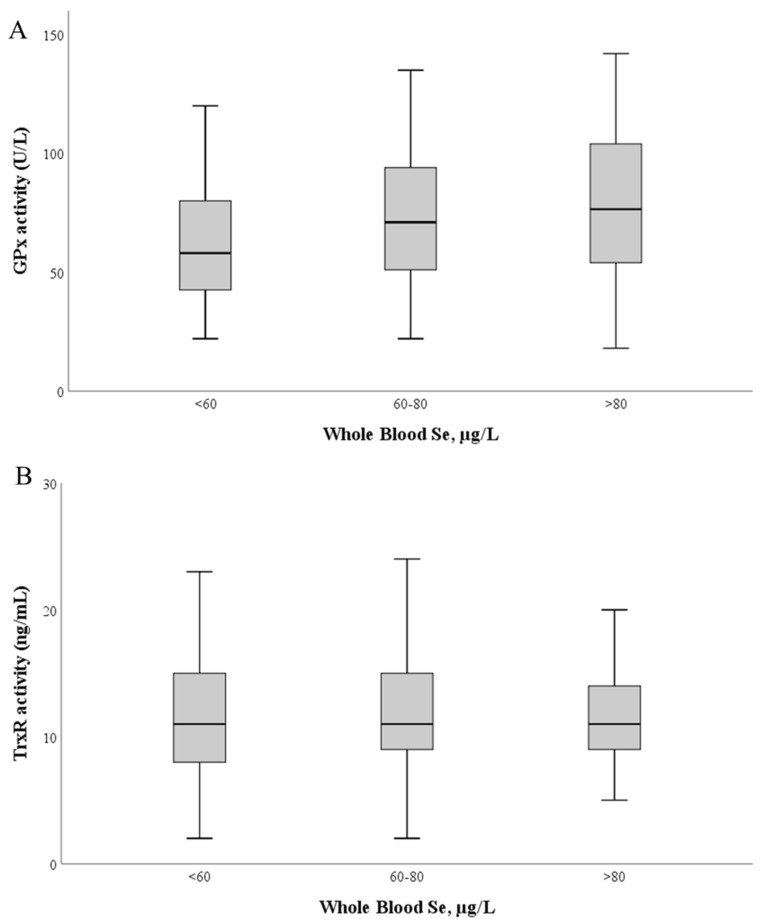
Glutathione peroxidase (**A**) and thioredoxin reductase (**B**) activities as a function of whole blood Se levels. The median value is represented as a bold line inside the box. Se—selenium; GPx—glutathione peroxidase; TrxR—thioredoxin reductase.

GPx activity (Figure 3) ranged between 17.9 and 155.1 U/L (mean 69.7 ± 28.4 U/L; median: 66.8 U/L). No correlation was found between GPx activity and Se levels in whole blood (r = 0.11; *p* = 0.111) or plasma (r = −0.89; 0.49) (Figure 3A).

TrxR activity ranged between 1.9 and 62.4 ng/mL (mean: 12.3 ± 5.6 ng/mL; median: 11.3 ng/mL). Similarly, no significant correlation was found between TrxR activity and Se levels in whole blood (r = 0.031; *p* = 0.50) and plasma (r = 0.090; *p* = 0.25) (Figure 3B).

**Figure 4:**
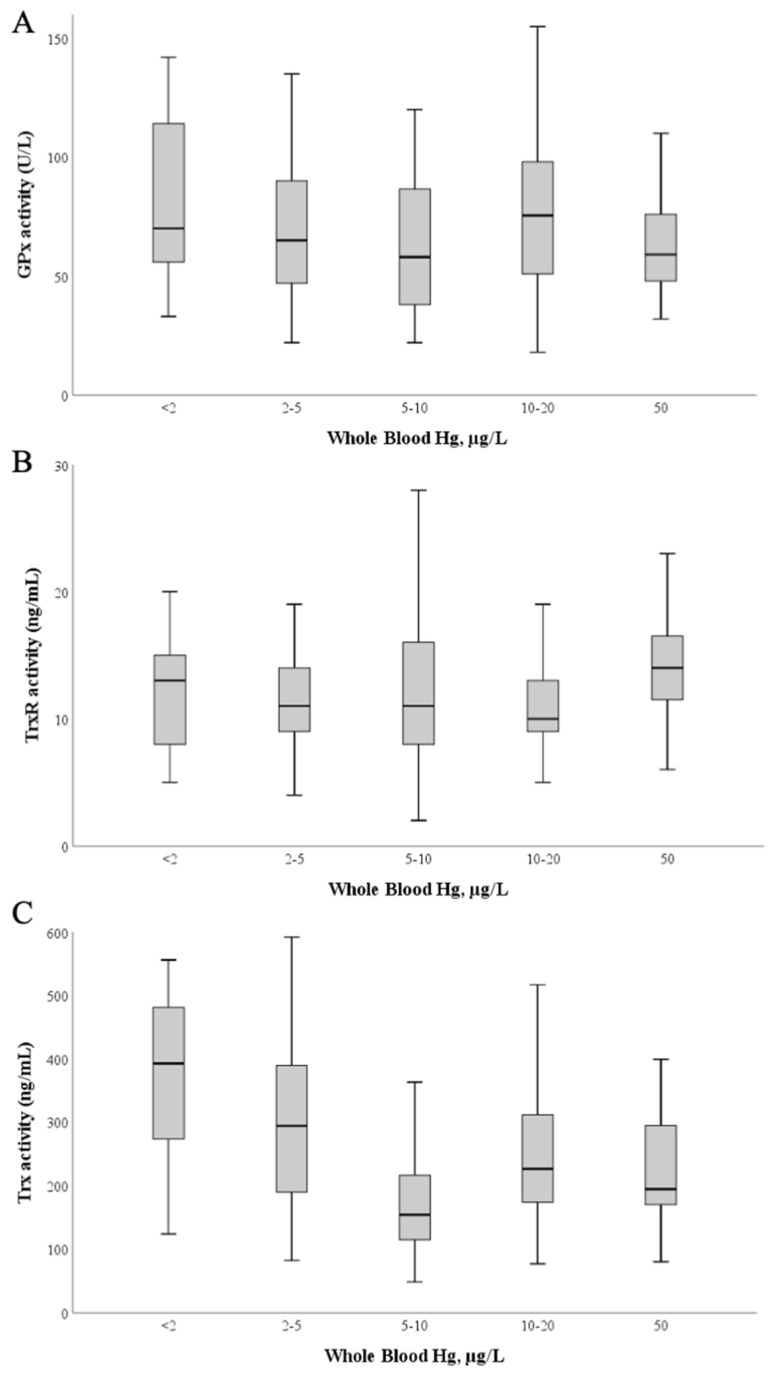
Glutathione peroxidase (**A**), Thioredoxin reductase (**B**) and Thioredoxin (**C**) activities as a function of blood Hg levels. The median value is represented as a bold line inside the box. Hg—mercury; GPx—glutathione peroxidase; TrxR—thioredoxin reductase; Trx—thioredoxin.

To understand if the high exposure to Hg of these pregnant women (previously reported in [50]) could influence the activity of these enzymes in plasma, we investigated the relation between activity levels and blood Hg (Figure 4). No significant correlations were found between enzymatic activities of GPx, TrxR and blood Hg levels (Figure 4A,B; *p* > 0.05).

Although Trx is not a selenoprotein, its activity was also evaluated as it integrates the thioredoxin system and has also been identified as a target of Hg [40]; Trx activity varied between 48 and 598 ng/mL, with a mean of 236 ± 120 ng/mL and a median value of 201 ng/mL. Interestingly, by applying simple linear regression calculations Table A1) (Appendix B) using blood Hg as the independent variable and Trx activity as the dependent variable, a significant model (*p* = 0.037) is found with a negative standardized regression coefficient between both variables (β = −0.096; *p* = 0.037), though the total explained variance is low (R^2^ = 0.009) (Figure 4C).

## 4. Discussion

The mean Se value in whole blood for the population of pregnant women from Madeira (65.0 ± 13.1 µg/L) is in general lower than values reported in different locations in Portugal (Table 2).

Interestingly, the first report of population Se levels in Portugal, which dates from 1986 [52] and was part of the Danish pilot study Euro–Selen 83, reported serum Se levels (101 ± 13 µg/L for males and 103 ± 7 µg/L for females) that were among the highest concentrations measured in several countries, which the authors attributed to Portuguese food habits, namely fish and seafood consumption. Indeed, fish consumption is among the dietary factors thought to contribute the most to a high blood Se level [56]. In agreement, another study in the Azores [54], analyzing Se levels in 433 individuals from 20 to 60 years and belonging to five populations of different socioeconomic classes and different dietary habits, found significant differences between men living in urban or rural areas compared with those living in coastal fishing communities, which had a higher fish intake and plasma Se (Table 1).

In our study, women reported a mean intake of 2.4 ± 1.4 fish meals per week with some women reporting up to 8 meals [51]. Thus, a higher value of blood and plasma Se would be expected in this population. However, the values for both whole blood and plasma were generally lower in pregnant women from Madeira than mean values reported elsewhere [52,53,55,57,58] (Table 2). For example, reference values established for the Spanish population are between 66.7 and 119.4 µg/L in whole blood [59].

A possible explanation for the lower Se levels found in this study may be related to the pregnancy status of the population being studied and the potentially inadequate Se intake. In fact, Se requirements during pregnancy increase 2 to 4 µg per day [7] and some authors have reported significantly lower Se values in pregnant women relatively to non-pregnant controls [60,61,62] (see Table 3), which is attributed to the transference of Se to the tissues of the developing fetus and also the dilution caused by the increase in blood volume of the mother [63]. Some studies that examined Se levels during pregnancy also found a tendency for lower blood/plasma levels as pregnancy progressed, however, our data do not show any significant (*p* > 0.05) change in Se levels from the 1st to the 3rd pregnancy trimester (Table 2) and thus, pregnancy alone cannot explain the lower levels observed. Therefore, an overall insufficient intake through the diet needs to be considered.

Moreover, it should be mentioned that this population has a high exposure to Hg as a result of fish consumption, especially a considerable intake of fresh tuna and black scabbard fish [51]. The interaction of Hg with Se has been linked to its detoxification mechanism but, on the other hand, it also leads to decreased Se bioavailability and inhibition of selenoproteins [22,23,28,38]. This mechanism could contribute to lower blood Se levels and justify the need to adjust Se intake for this population, via supplementation or dietary modifications such as an increase in the intake of Se rich foods (e.g., nuts). This approach is also supported by several reports indicating that low Se levels in women may be associated with complications during pregnancy [63].

Plasma GPx activity found in pregnant women from the island of Madeira (69.7 ± 28.4 U/L) is lower than reported for other healthy populations [62,64,65]. This is not surprising given the relatively low levels of Se found in this study. In fact, plasma Se levels found in this study (46.1 ± 11.1 µg/L) are below the value set by the WHO (63.2 µg/L) to optimize plasma GPx activity [7]. Indeed, it is known that plasma GPx (GPx3) activity is a good indicator of Se status [66] since upon sub-optimal Se intake, incorporation of Se is directed towards the synthesis of selenoproteins ranking higher in hierarchy [67], leading to a decrease in GPx3 levels (Figure 1). 

Unlike plasma GPx, TrxR plasma levels (mean: 12.3 ± 5.6 ng/mL: median: 11.3 ng/mL) in our study population agree with levels found in healthy subjects in other studies [45,68]. Interestingly, TrxR levels did not show a relation to Se levels (Figure 3B). This is likely due to TrxR, contrary to GPx3, being a high priority selenoprotein with housekeeping functions, which prompts maintenance of basal levels even at sub-optimal Se exposure [67,69] (Figure 1). 

**Figure 5:**
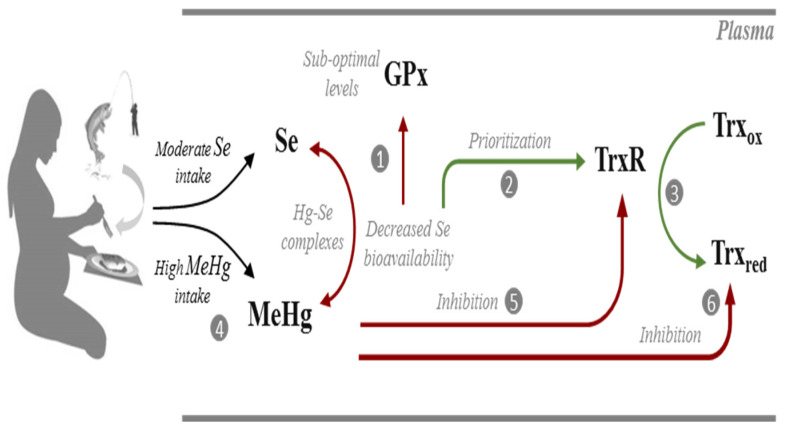
Possible interactions occurring in the plasma of pregnant women from Madeira between selenium (Se), selenoproteins (GPx; TrxR), methylmercury (MeHg) and thioredoxin (Trx). At suboptimal intake levels, Se bioavailability is not sufficient to optimize GPx activity (1). Instead, Se is channeled to the synthesis of TrxR which is a high priority selenoenzyme (2) allowing efficient reduction of Trx (3). Due to the significant consumption of predatory fish species, these pregnant women have a high exposure to MeHg (4) which could potentially form complexes with Se and decrease its bioavailability. MeHg may also target TrxR (5) but activity levels are maintained via the prioritization of its synthesis in the face of sub-optimal Se levels. Albeit TrxR activity is optimized, the activity of Trx is reduced due to the direct interaction with MeHg (6). Biochemical pathways are simplified for clarity. Red lines indicate sub-optimized pathways or an inhibitory interaction. Green lines indicate optimized pathways.

TrxR, as a result of its active site structure and C-terminal location of the selenocysteine residue, has a broad substrate specificity [70]. However, this molecular promiscuity also renders TrxR susceptible to electrophiles such as Hg compounds [22]. Thus, in the samples of pregnant women from the island of Madeira, we anticipated that the high exposure to MeHg of this population could lead to lower plasma TrxR. Surprisingly, we did not see such effect even for subjects with high blood Hg levels (Figure 4B). It could be that the available Se is enough to allow adequate levels of de novo expression of TrxR to keep its activity even in the presence of Hg compounds (Figure 5). This has been observed previously in cell experiments, where co-exposure to Hg compounds and sodium selenite could prevent TrxR1 (cytosolic isoform) inhibition [71]. 

Interestingly, Trx activity tended to decrease with increasing Hg values (Figure 4C). Thioredoxin 1, the main substrate of TrxR1, is a 12 kDa protein containing five Cys residues in its structure and is a known target for Hg compounds [37,40], and its inhibition ultimately leads to cell death [72]. Moreover, in vitro and in vivo experiments showed that, unlike that verified with TrxR, the inhibition of Trx by MeHg occurs independently of Se status [38,71]. Indeed, MeHg is known to cause the oxidation of all of Trx’s Cys residues either by direct interaction or due to ROS formation, including structural Cys 63, 69 and 73 residues which are not targets for TrxR [40,72]. 

Thus, in this population, the decreasing tendency of Trx activity with exposure to Hg seems to reflect a toxic effect which cannot be prevented by nutritional factors, i.e., available Se (Figure 5). However, given the variability in Trx levels within each Hg exposure level, the utility of Trx activity as a possible biomarker of effect for Hg is limited. Nonetheless, Trx activity deserves further monitoring in future epidemiologic surveys. 

Plasma TrxR and Trx derive from intracellular pools. Existing evidence has shown that exposure to mercurials induces Nrf-2 mediated transcription of TrxR1 [71]. However, this mechanism does not seem to have an impact on TrxR activity in plasma because either the secretion of TrxR/Trx into plasma is not occurring or it is masked by the inhibition of these enzymes by mercurials. Since both the cytosolic and mitochondrial isoforms of TrxR and Trx are targeted by mercury [71,72], an analysis of whole-blood activities might give a more refined look at the effect of Hg exposure. However, evaluation of TrxR/Trx activity in blood is technically difficult since hemoglobin interferes with DTNB detection and assay sensitivity.

Overall, the results of this study show that in Madeira, (Portugal) Se levels in whole blood and plasma samples of pregnant women are below recommended values. Albeit we did not quantify Se intake, this finding was surprising given the dietary habits of the population under study, which include a high intake of fish and seafood. However, this can also explain the low Se levels since fish, in particular the predatory fish these women consume, is a source of MeHg which could decrease Se bioavailability. Despite this, the effect of the lack selenium was not evident in selenoproteins’ activity. On the other hand, the decrease in activity observed in Trx indicates a molecular interaction with MeHg (Figure 5). 

A more powerfully designed study cohort (longitudinal) would be useful to better understand the impact of Se status on Hg-selenoprotein interaction, and how it reflects in fetal health could be studied by looking at these end-points also in cord-blood/plasma. Additionally, analysis of the oxidative stress index would be a valuable tool to complement these results.

In spite of these limitations, we can conclude that low Se levels are reflected in a lower plasma activity of GPx, which agrees with its low priority level in selenoprotein synthesis. In line with this, TrxR activity levels are within expected levels since, due to its prioritization, available Se will be channeled to its synthesis. Exposure to Hg did not show a direct impact on selenoprotein activity but may contribute to reduce Se bioavailability. Most importantly, the TrxR-dependent enzyme, thioredoxin, showed a significant decrease in activity as Hg levels increased, showing a possible impact on the redox status of these women.

In light of these results, it would be important to: (1) promote the consumption of Se rich foods or supplement intake by pregnant women; (2) evaluate how Se levels and enzymatic activities in the fetus reflect the observations in maternal blood/plasma; and (3) consider the effect of co-exposure to MeHg on Se, selenoproteins and Trx activity in populations presenting high consumption of predatory fish. Indeed, just assessing blood or plasma Se levels is an oversight on the overall selenium status. The low Se status associated with a considerable intake of MeHg could magnify the probability and severity of adverse outcomes in an already high-risk population. 

## Figures and Tables

**Table 1:** Population characteristics for the pregnant women enrolled in the study (N = 474 *).

Age (years)	29 ± 6	
Gestational weight (Kg)	67 ± 12	
Height (cm)	165 ± 70	
Week of Gestation **	20 ± 10	
Seafood eating habits (N)	Yes	428
	No	34
	DK/DA	12

* Number of women that responded to the questionnaire; ** At the moment of sample collection; DK/DA (do not know or do not reply).

**Table 2:** Selenium levels in whole blood (^WB^) and/or plasma/serum (^P^) in several studies with the Portuguese population.

Gender (N)		Selenium (µg/L)		Location	Reference
Male	Female	Male	Female		
-	513 167	-	65.0 ± 13.1 ^WB^ 46.1± 11.1 ^P^	Madeira	Present study
14	13	101 ± 1 ^P^	103 ± 7 ^P^	Not specified	[52]
136	47	88 ± 15 ^P^	81 ± 14 ^P^	Lisbon (urban)	[53]
48	50	98 ± 16 ^P^	81 ± 14 ^P^	Azores, Ponta Delgada (urban)	[54]
26	49	110 ± 25 ^P^	90 ± 21 ^P^	Azores, Rabo de Peixe (fishing)	
28	44	84 ± 22 ^P^	78 ± 18 ^P^	Azores, Rabo de Peixe (rural)	
28	71	90 ± 20 ^P^	82 ± 17 ^P^	Azores, Água Retorta (rural)	
26	63	108 ± 16 ^P^	96 ± 23 ^P^	Ribeira Quente (fishing)	
62	39	99.0 ± 20.5 ^P^	94.4 ± 16.7 ^P^	Lisbon (urban)	[55]
48	50	97.9 ± 16.2 ^P^	88.1 ± 14.5 ^P^	Azores (urban)	
16	19	84.5 ± 16.5 ^P^	84.2 ± 14.4 ^P^	Salvaterra de Magos (rural)	

**Table 3:** Selenium levels in whole blood ^(WB)^ and/or plasma/serum ^(P)^ in studies with pregnant women.

Women (N)				Selenium (µg/L)				Reference
Trimester			Not Pregnant	Trimester			Not Pregnant	
1	2	3		1	2	3		
171 *	81 *	84 *	-	65.0 ± 11.2 *	65.3 ± 13.1 *	63.0 ± 9.1 *	-	This study
48	48	50	74	64.2 ± 16.9 ^P^	66.6 ± 17.1 ^P^	61.6 ± 15.0 ^P^	70.7	[60]
34	44	52	30	107 ± 16.0 ^P^	83.0 ± 17.4 ^P^	79.8 ± 19.0 ^P^	109 ± 14.2 ^P^	[61]
49	38	30	22	75.8 ± 16.7 ^WB^	65.6 ± 14.0 ^WB^	55.2 ± 12.5 ^WB^	95 ± 9.3	[62]

* mean values were calculated including the number of women that reported the weeks of gestation.

**Figure A2:**
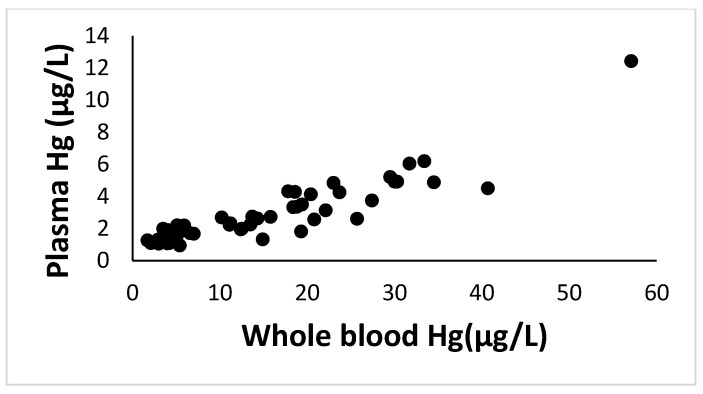
Mercury (Hg) levels (µg/L) in whole blood versus levels in plasma. A significant correlation (Spearman Correlation rank: 0.90; *p* < 0.001) was found between Hg in blood and plasma over a broad range of Hg levels and thus Hg in whole blood was used to evaluate relationships with enzymatic activities since the number of plasma samples available for Hg analysis was limited.

## Data Availability

Data are available on request (corresponding author).

## Appendix B

**Table A1:** **Summary** Statistical parameters of a simple linear regression between blood Hg levels (independent variable) and Trx activity in plasma (dependent variable).

**Variables Entered/Removed ^a^**							
Model		Variables Entered		Variables Removed	Method		
1		Hg (µg/L) WB ^b^			Enter		
a. Dependent Variable: Trx (ng/mL) Plasma							
b. All requested variables entered.							
**Model Summary**							
Model		R	R Square	Adjusted R Square	Std. Error of the Estimate		
1		0.096 ^a^	0.009	0.007	119.71437		
a. Predictors: (Constant), Hg (µg/L) WB							
**ANOVA ^a^**							
Model		Sum of Squares		df	Mean Square	F	Sig.
1	Regression	62822.470		1	62822.470	4.384	0.037 ^b^
	Residual	6721487.713		469	14331.530		
	Total	6784310.183		470			
a. Dependent Variable: Trx (ng/mL) Plasma							
b. Predictors: (Constant), Hg (µg/L) WB							
**Coefficients ^a^**							
Model		Unstandardized Coefficients			Standardized Coefficients	t	Sig.
		B		Std. Error	Beta		
1	(Constant)	251.551		9.196		27.353	0.000
	Hg (µg/L) WB	−1.733		0.828	−0.096	−2.094	0.037
a. Dependent Variable: Trx (ng/mL) Plasma							
